# Immune Protective Effect of Chitosan Oligosaccharide on Lipopolysaccharide-Stimulated Coelomocytes of Sea Cucumber *Apostichopus japonicus* In Vitro

**DOI:** 10.3390/polym17202752

**Published:** 2025-10-14

**Authors:** Rongyue Wang, Xiaoyu Nie, Xiaofan Li, Jinwei Tang, Chong Huang, Juan Liu

**Affiliations:** College of Fisheries and Life Science, Dalian Ocean University, Dalian 116023, China; wangrongyue97@icloud.com (R.W.); niexiaoyu1001@163.com (X.N.); lixiaofan1921@163.com (X.L.); a18712841320@163.com (J.T.); 13292713055@163.com (C.H.)

**Keywords:** chitosan oligosaccharide, *Apostichopus japonicus*, immunoprotection, lipopolysaccharide, coelomocytes, transcriptomic analysis

## Abstract

In recent years, chitosan oligosaccharide (COS) has demonstrated promising applications in enhancing the immune protective function of sea cucumbers. However, the immune-protective effect of COS on sea cucumber coelomocytes in vitro remains unclear. This study investigated the effect of COS on lipopolysaccharide (LPS)-stimulated inflammation in sea cucumber coelomocytes. First, we measured the effects of COS and LPS on the viability of coelomocytes. COS exhibited no toxic effects on sea cucumber coelomocytes. Furthermore, pre-incubating the coelomocytes with COS significantly improved coelomocytes’ viability after LPS stimulation (*p* < 0.05). Secondly, the phagocytic activity and respiratory burst of the coelomocytes were assessed to evaluate their immune levels. COS alone significantly increased the respiratory burst and phagocytic activity of the coelomocytes (*p* < 0.05). However, with LPS stimulated, COS significantly increased both the respiratory burst and phagocytic activity of the coelomocytes. The activities of lysozyme (Lyz), total nitric oxide synthase (T-NOSs), and superoxide dismutase (SOD) in sea cucumber coelomocytes were measured to evaluate their response to LPS stimulation. The results indicated that LPS stimulation significantly increases the activities of Lyz, T-NOSs, and SOD in sea cucumber coelomocytes (*p* < 0.05). Additionally, it was found that COS could inhibit the LPS-mediated expression of Lyz, T-NOSs, and SOD activities in the coelomocytes (*p* < 0.05). Furthermore, the relative expression of six immune-related genes—*Aj-IL-17*, *Aj-TNF-α*, *Aj-i-Lys*, *Aj-NOS*, *Aj-Rel*, *Aj-P105*—were analyzed in the coelomocytes stimulated by LPS after being cultured with COS. Finally, through transcriptomic technology analysis, it was determined that COS primarily alleviates LPS-induced inflammation via the tumor necrosis factor signaling pathway and the phagosome signaling pathway. The findings demonstrated that COS inhibited the expression of immune genes in sea cucumber coelomocytes in a dose-dependent manner. In summary, pretreatment with chitosan oligosaccharides appears to confer an immune protective role in LPS-stimulated sea cucumber coelomocytes.

## 1. Introduction

The sea cucumber *Apostichopus japonicus*, which belongs to the phylum Echinodermata and the class Holothuroidea, is a fishery species of significant economic importance in Asian countries [1]. However, because of ecological imbalance, environmental pollution, and the expansion of farming, worldwide sea cucumber culture has been threatened by various serious bacterial diseases, especially the skin ulceration syndrome (SUS) [2]. It was reported that the potential pathogens causing SUS might be Gram-negative bacteria [3]. As the main component of the cell wall of Gram-negative bacteria, LPS was proved to be able to induce significant immune responses in sea cucumbers and could directly cause cellular injury, dysfunction, and death [4,5,6]. Sea cucumbers lack an adaptive immune system and rely on their innate immune system, which mainly includes cellular immunity and humoral immunity, to resist and eliminate invading pathogens [7]. The cellular immune system of sea cucumbers is composed of various coelomocytes. These cells, found in the body cavity fluid, secrete a range of immune factors that contribute to the humoral immune system. Cellular immunity and humoral immunity work together to supplement the immune function of sea cucumbers [8]. In cellular immunity, coelomocytes defend against pathogen infection by modulating some regulator elements, expressions of immune-related genes, and activations of immune-related signaling pathways [9]. The humoral immune response is mediated by the secretion of various immune factors into the coelomic cavity by coelomocytes. These factors include lectins, enzymes, lysozyme, Toll receptors, and inflammatory mediators [10]. Generally, the immune factors of sea cucumbers serve as the primary receptors in the host defense against pathogenic bacterial invasion [11]. Therefore, it is urgent and necessary to find a non-toxic, green substance to solve the damage in sea cucumber culture. Recently, numerous natural compounds have been discovered to possess potent anti-inflammatory activity and have attracted considerable interest [12,13,14]. Due to the safety profile and potentially beneficial biological activities of natural products, there is a new focus on the search for natural ingredients with immunoregulatory and anti-inflammatory properties to prevent or treat this inflammation.

**Figure 1:**
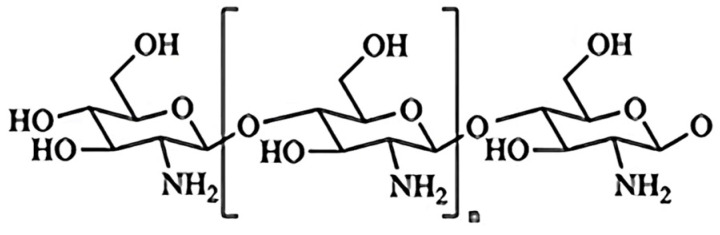
Chemical structure of Chitosan Oligosaccharides.

Chitosan oligosaccharides (COS) are oligomers of β(1–4)-linked *N*-acetylglucosamine and D-glucosamine and are produced from chitin or chitosan using different enzymatic or chemical methods [15]. It exhibits distinctive biological activities such as antioxidant, anti-fungal, antibacterial, and anti-tumor activity [16]. COS exhibit a smaller molecular weight (MW), a lower degree of polymerization (DP), and enhanced water solubility compared to chitosan [17]. The chemical structural formula of COS is shown in Figure 1 [18]. In recent years, COS have garnered increasing interest due to their anti-inflammatory and immunological properties, with most studies focusing on mammalian subjects, yielding intriguing results. For example, COS reduced the signals induced by lipopolysaccharides (LPS) and the production of inflammatory mediators in IPEC-J2 cells [19]. Furthermore, COS effectively inhibited the phosphorylation of IκB, JNK, and P65, ultimately leading to a decrease in macrophage inflammatory response in mice [20]. Although the application of COS is common in mammalian cells, it has not been researched deeply in sea cucumber coelomocytes.

With the advancement of high-throughput sequencing technology, this technique has been increasingly applied in the research of various aquatic animals, including *Takifugu rubripes* [21], the pearl oyster [22], the sea urchin [23], *Litopenaeus vannamei* [24], and the sea cucumber [25]. This technology is particularly useful for studying sea cucumber diseases, immunity, growth, stress resistance, and metabolic mechanisms. Transcriptomic analysis of *Apostichopus japonicus* challenged with *Vibrio splendidus* revealed significant changes in gene expression across various tissues, particularly in the body wall, which exhibited a stable up-regulation of genes associated with immune responses, oxidative stress, and apoptosis during the progression of skin ulceration syndrome [26]. The identification of differentially expressed genes (DEGs) in response to low-salinity stress revealed pathways involved in carbohydrate metabolism, fatty acid degradation, and amino acid metabolism, highlighting the metabolic adaptations that enable sea cucumbers to cope with osmotic stress [27]. Differential gene expression analysis between fast-growing and slow-growing individuals of *Apostichopus japonicus* revealed a significant down-regulation of genes associated with ribosome biogenesis and protein synthesis [25].

In this study, we investigated differential responses of phagocytic activity and respiratory burst activity, the activities of three immune-related enzymes such as Lysozyme (Lyz), Total Nitric Oxide Synthase (T-NOSs), and superoxide dismutase (SOD), and the expression patterns of six immune indicators—*Aj-IL-17*, *Aj-TNF-α*, *Aj-i-Lys*, *Aj-NOS*, *Aj-Rel*, *Aj-P105*—from the sea cucumber coelomocytes after COS pretreatment and LPS challenge. To understand the immune mechanisms of coelomocytes in *Apostichopus japonicus* stimulated by LPS, we sequenced the transcripts of coelomocytes under LPS stimulation using the Illumina HiSeq™ 2000 platform. The differentially expressed genes (DEGs) were annotated through Gene Ontology (GO) functional analysis, and Kyoto Encyclopedia of Genes and Genomes (KEGG) pathway analysis. Several DEGs were randomly selected for validation to identify the genes involved in the immune response and to explore the molecular immunoprophylactic mechanisms of COS. This study offers new insights into the immune protective effects of COS on the inflammation of coelomocytes in *Apostichopus japonicus* induced by LPS stimulation, providing comprehensive data for a better understanding of the innate immune response in *Apostichopus japonicus*.

## 2. Materials and Methods

### 2.1. Chemicals and Reagents

Chitosan oligosaccharides were provided by the Natural Products and Sugar Engineering Group of the Dalian Institute of Chemical Physics, Chinese Academy of Sciences. The team’s research showed that the deacetylation degree of COS was determined to be 88% by ‘H NMR spectroscopy [28]. The polymerization degrees of COS were 2–6, the percentages of which were identified by HPLC to be 8.75 ± 0.50%, 22.68 ± 0.60%, 32.32 ± 0.60%, 24.92 ± 0.43, and 11.33 ± 0.25% [29]. L-15 was purchased from TransGen Biotech (Beijing, China), and fetal bovine serum (FBS) was purchased from Sigma (Saint Louis, MO, USA). Enzyme activity kits were obtained from Nanjing Jiancheng (Nanjing, China). A Mona Real-time quantitative PCR kit was used for RT PCR, and all primers were synthesized by Shanghai Sangon Bioengineering Co., Ltd. (Shanghai, China).

### 2.2. Experimental Animals and Primary Culture Coelomocytes of Sea Cucumber

Healthy adult sea cucumbers, with a body weight of 90.15 ± 6.67 g, were purchased from Dalian Baofa Treasure Co., Ltd. (Dalian, China). They were dispersed immediately into 40-L tanks filled with aerated seawater with a salinity of 30 at darkroom temperature (15–18 °C) for at least 3 days before the experiment. The coelomic fluid was withdrawn from the coelom of sea cucumbers using sterile syringes. An equal volume of anticoagulant solution (0.02 M EDTA, 2.8% NaCl, 0.019 M KCl, 0.068 M Tris-HCl, pH 6) was then added to the collected fluid. The resulting mixture of coelomic fluid and anticoagulants was centrifuged at 800× *g* for 5 min at 16 °C. The supernatant was discarded, and the cell pellet was collected. Subsequently, the cells were washed twice with an isotonic buffer. (0.001 M EGTA, 0.53 M NaCl, and 0.01 M Tris-HCl, pH 7.6). The cell was resuspended in Leibovitz’s L-15 with 10% FBS and a penicillin-streptomycin solution (100 mg/mL streptomy-cin and 100 U/mL penicillin) to obtain a final cell concentration of 1 × 10^6^ cells ml^−1^.The cells were incubated at 18 °C in a constant-temperature biochemical incubator, and cell morphology and proliferation were observed daily using an inverted microscope (Olympus Corporation, IX51, Tokyo, Japan). Aliquots of 200 µL cell suspension were dispensed into the wells of 96-well culture microplates for the cell viablility assay, phagocytic activity assay, and respiratory burst activity assay. Aliquots of 1 mL cell suspension were dispensed into the wells of 24-well culture microplates for enzyme activity assay. Aliquots of 2 mL cell suspension were dispensed into the wells of six-well culture microplates for quantitative reverse transcription-PCR (qRT-PCR) analysis assay. The coelomocytes were incubated at 18 °C overnight in darkness prior to immune stimulant adding.

### 2.3. Cell Viablility Assay

The CCK-8 assay was utilized to explore the impact of different levels of COS (0, 50, 100, 200, and 400 μg/mL) on the viability of coelomocytes. Coelomocytes were cultured overnight in 96-well cell plates with a density of 1 × 10^5^ cells per well before treatment. Following this, the cells were exposed to varying concentrations (0, 50, 100, 200, and 400 μg/mL) of COS for 6, 12, and 24 h. Subsequently, 10 μL of CCK-8 solution was added to each well containing 100 ul of coelomocytes to assess cell viability at 18 °C for 1 h. Absorbance was determined at 450 nm using a 96-well plate reader using an EL 800 Absorbance Microplate Reader (BioTek Instruments, Inc., Winooski, VT, USA). Each concentration was repeated three times in the well.

### 2.4. Phagocytic Activity

The cells were processed in the same way as above. Then, the phagocytosis activity was determined by the neutral red staining method [30]. Briefly, the supernatant was carefully discarded, and a neutral red solution (0.05% of the neutral red stock in cell culture medium without serum) was added to each well, and the cells were incubated for 30 min. At the end of the incubation period, the neutral red solution was removed, and the wells were washed with PBS twice. Subsequently, a fixative solution (100 μL) was added to each well, and absorbance was measured at 540 nm on a plate reader.

### 2.5. Determination of Enzyme Activity

Coelomocytes were cultured overnight in 12-well cell plates with a density of 1 × 10^5^ cells per well before treatment. Following this, 6 h after stimulation with varying concentrations (100 and 200 μg/mL) of COS, coelomocytes were treated with LPS (1 μg/mL) for another 6 h. The supernatant was carefully discarded, and the pellet was resuspended in an amount of PBS. The cells were then broken by ultrasonic cell disruption. Subsequently, enzyme activity was detected following the kit instructions (Nanjing Jiancheng, Nanjing, China). The enzyme activities of Superoxide Dismutase (SOD), Lysozyme (Lyz), and Total Nitric Oxide Synthase (T-NOSs) were determined. SOD activity was determined by the hydroxylamine method (A001-1-2), Lyz activity was determined by the turbidimetric method (A051-1-1), and T-NOSs activity was determined by the colorimetric method (A014-2-1).

### 2.6. Respiratory Burst Activity

The cells were processed in the same way as above. The experimental method for detecting respiratory burst activity was based on methods described in the literature with some modifications [31]. The enzyme plate is centrifuged first, discarding the supernatant. Following that step, 200 mL of 0.2% NBT solution should be added and incubated for 30 min. Centrifuge again, discard the supernatant, and proceed by adding 200 mL of 70% methanol to fix the sea cucumber coelomocytes. Wash the cells twice with a 70% methanol solution and air-dry the 96-well microplate. Post air-drying, 120 μL of 2 mol KOH solution should be added to each well to lyse the sea cucumber coelomocytes, followed by the addition of 140 μL of dimethyl sulfoxide (DMSO) to dissolve the blue formazan formed in the cytoplasm. The 96-well microplate should then be placed on a microplate reader and shaken slowly for 5 min to ensure the even mixing of the solutions in the microwells. Subsequently, the absorbance value should be read at room temperature using a microplate reader at a wavelength of 620 nm.

### 2.7. Total RNA Extraction and Quantitative Reverse Transcription-PCR (qRT-PCR) Analysis

Coelomocytes (4 × 10^6^ cells/mL) were cultured overnight in six-well plates and treated with LPS and COS for 6 h. Following treatment, the culture medium was discarded, and cells were washed with PBS, collected into sterile centrifuge tubes, and centrifuged at 4 °C 800× *g* for 5 min. The supernatant was then removed, and the cell pellet was treated with 1 mL of Trizol reagent (Sigma) for total RNA extraction. The integrity and purity of the RNA were confirmed using 1% agarose gel electrophoresis and UV spectrophotometry. The OD_260_/OD_280_ ratio was higher than 1.8, making reverse transcription possible. The extracted RNA was further purified by treating it with DNase I to remove genomic DNA. Subsequently, RNA was reverse transcribed into cDNA using the PrimeScript™RT reagent Kit (Takara, Tokyo, Japan). The cDNA was then utilized for quantitative real-time RT-PCR analysis on an ABI Step One Plus System using the SYBR Premix Ex Taq II Kit (Takara). To assess gene expression stability, β-actin was used as a reference gene. The relative expressions of the target genes, namely *Aj-NOS*, *Aj-i-Lys*, *Aj-TNF-α*, *Aj-IL-17*, *Aj-Rel*, and *Aj-P105*, were analyzed by the 2^−ΔΔCt^ method. The following reaction mixture for each sample was used: 1 μL cDNA, 0.8 μL of primers at the final concentration of 0.2 μM, 10 μL 2XSYBR Green Pro Taq HS Premix, 0.4 μL ROX Reference Dye, and 7.8 μL RNase-free water. The qPCR reaction program consisted of initial denaturation at 95 °C for 10 min, followed by 40 cycles at 95 °C for 10 s, 60 °C for 10 s, and 72 °C for 30 s. The primer sequences used in this study are listed in Table 1. The mean level of relative expression ± SE from three biological replicates represents the transcript levels.

### 2.8. Library Preparation and Sequencing

A total of six samples are necessary for the construction of the library (LPS group: coelomocytes were stimulated solely with LPS; EG group: coelomocytes were initially stimulated with COS, followed by LPS stimulation, *n* = 3). An input of 1 μg RNA per sample was utilized for the preparation of RNA samples. Sequencing libraries were created using the VAHTSTM mRNA-seq V2 Library Prep Kit for Illumina^®^ (Vazyme Biotech Co., Ltd., Nanjing, China), adhering to the manufacturer’s guidelines, and index codes were incorporated to associate sequences with each sample. In brief, mRNA was isolated from total RNA using poly-T oligo-coated magnetic beads. Fragmentation was achieved using divalent cations at an elevated temperature in the VAHTSTM First Strand Synthesis Reaction Buffer (5×). The first strand of cDNA was generated using random hexamer primers and M-MuLV Reverse Transcriptase (RNase H-). Following this, the synthesis of the second strand of cDNA was conducted with DNA polymerase I and RNase H. Any remaining overhangs were converted to blunt ends through exonuclease/polymerase activity. After adenylating the 3′ ends of the DNA fragments, an adaptor was ligated for library preparation. To selectively retrieve cDNA fragments of approximately 150–200 bp in length, the library fragments were purified using the AMPure XP system (Beckman Coulter, Beverly, CA, USA). Subsequently, 3 μL of USER Enzyme (NEB, Ipswich, MA, USA) was employed with the size-selected, adaptor-ligated cDNA at 37 °C for 15 min, followed by 5 min at 95 °C before proceeding with PCR. PCR was carried out using Phusion High-Fidelity DNA polymerase along with Universal primers and an Index (X) Primer. Ultimately, PCR products were purified (using the AMPure XP system), and the library’s quality was evaluated on the Agilent Bioanalyzer 2100 system. The libraries were then quantified and pooled, followed by paired-end sequencing conducted on the NovaSeq sequencers (Illumina, San Diego, CA, USA).

### 2.9. Sequencing Data Quality Control

The raw data quality of the samples was initially analyzed using the FastQC (version 0.11.2) software. Based on the quality assessment report, the Trimmomatic tool was employed to perform data cleaning, removing adapter sequences, N bases, and base segments with quality scores below 20, to obtain clean reads. Subsequently, quality metrics of the processed sequencing data, including Q20, Q30 values, and GC content, were analyzed. The reference genome sequence of *Apostichopus japonicus* was obtained from the NCBI database (https://www.ncbi.nlm.nih.gov/datasets/genome/GCA_002754855.1/, accessed on 9 August 2024). The quality-controlled sequencing data were aligned to the reference genome using the HISAT2 (version 2.1.0) and RSeQC (version 2.6.1) software, followed by statistical analysis of the alignment results.

### 2.10. Differential Expression Gene Analysis and Functional Enrichment Analysis

This study employed the DESeq algorithm to conduct differential analysis of gene expression data. By setting stringent screening thresholds, the criteria for identifying significantly differentially expressed genes were defined as log2(FC) > 1.25 and adjusted *p* < 0.05. Using the clusterProfiler (version 3.6.0) software, GO functional enrichment analysis was performed on the differentially expressed genes in the coelomocytes of *Apostichopus japonicus*, with the top 10 most significant terms for each function selected to create bar charts. Additionally, KEGG pathway enrichment analysis was conducted on the differentially expressed genes screened from the coelomocytes of *Apostichopus japonicus* using the clusterProfiler software, and the top 20 most significant signaling pathways were selected for visualization.

### 2.11. Statistical Analyses

Results are shown as means ± SD. Student’s *t*-test was executed for comparison between two groups. *p* < 0.05 was defined as statistically significant. Statistical analyses were carried out using SPSS 20.0 software (IBM, Armonk, NY, USA).

## 3. Results

### 3.1. The Effects of COS on Cell Viability

**Figure 2:**
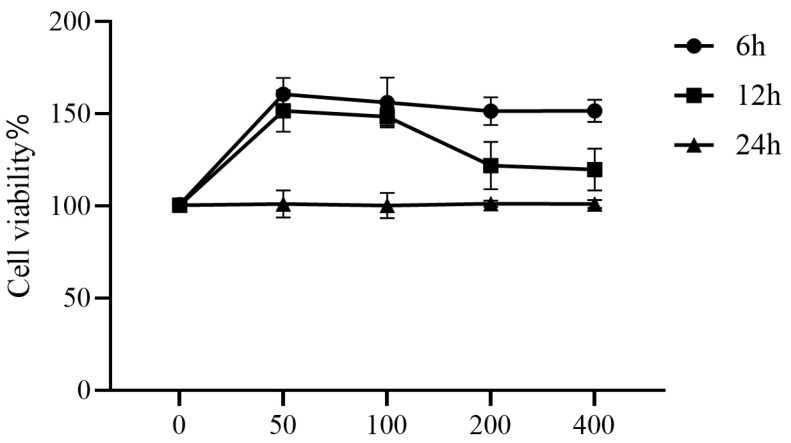
The effects of COS at different concentrations (50, 100, 200, and 400 μg/mL) on the viability of coelomocytes were observed after they had been treated for 6, 12, and 24 h, respectively. The Y-axis showed the percentage of cell survival, and the X-axis showed the doses of COS. Four biological replicates were performed in the experiment, and obtained data were expressed as the mean ± SD (*n* = 3).

In order to investigate the toxic effect of COS on coelomocytes, CCK-8 assay was used. Coelomocytes were cultured in 96-well culture plates with increasing concentrations of COS (0, 50, 100, 200, 400 μg/mL) for 6, 12, and 24 h in L-15 cell culture medium. The results for CCK8 showed that, compared with the control group, when the cells were treated with a 50, 100, 200, and 400 μg/mL concentration of COS, whether for 6 h or 12 h, the cell viability increased significantly (*p* < 0.05). After 24 h of treatment, the activity of cells treated with 50, 100, 200, and 400 μg/mL COS did not change significantly (*p* > 0.05), and the percentage of viable cells was 100.48%, 101.09%, 101.21%, and 101.77%, respectively (Figure 2). According to the test results for CCK8, COS had no toxic effect on sea cucumber coelomocytes in the range of 0–400 μg/mL. Additionally, for COS-treated coelomocytes, the percentage of viable cells was the highest at this concentration range at 6 h. Therefore, we chose 6 h as the best processing time.

### 3.2. Establishment of LPS-Stimulated Inflammation Model

**Figure 3:**
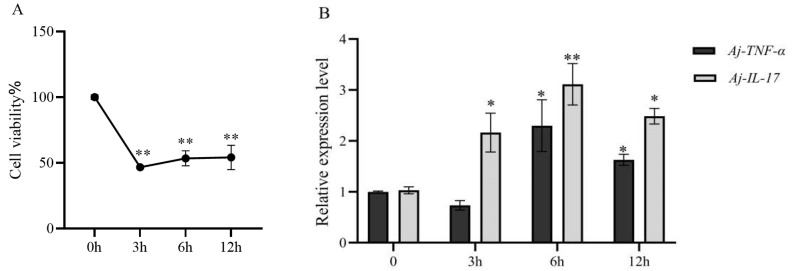
The effects of LPS on the viability of coelomocytes were observed after they had been treated for 3, 6, and 12 h, respectively. The Y-axis shows the percentage of cell survival, and the X-axis shows the different times (**A**). The relative fold changes of *AjIL-17* and *Aj-TNF-α* genes expressed in *A*. *japonicus* coelomocytes at 0 h, 4 h, 6 h, and 12 h after LPS challenge (**B**). * *p* < 0.05, ** *p* < 0.01 when compared to the LPS alone group. Data are expressed as the means ± SD (*n* = 3).

In order to investigate the effect of LPS on coelomocytes, CCK-8 assay and quantitative reverse transcription-PCR analysis were used. According to the results shown in Figure 3A, compared with control group, the viability of coelomocytes decreased by at least 53.35%, 47.17%, and 46.43% in the LPS-treated coelomocytes at 3 h, 6 h, and 12 h, respectively (*p* < 0.05). The secretion of pro-inflammatory cytokines in LPS-induced coelomocytes was also examined. For the *Aj-IL-17* gene, the relative expression of the gene was significantly increased at 3, 6, and 12 h after the cells were stimulated with LPS (*p* < 0.05). For the *Aj-TNF-α* gene, the relative expression of the gene was significantly increased after LPS stimulation for 6 h (*p* < 0.05). The relative expression levels of *Aj-IL-17* and *Aj-TNF-α* in coelomocytes stimulated with LPS (1 μg/mL) significantly increased at 6 h compared to the 4 h (*p* < 0.05) and 12 h (*p* < 0.05) groups., which indicates that the model of inflammation was successfully established in vitro (Figure 3B). Based on this result, we used 6 h LPS-stimulation for the subsequent experiments.

### 3.3. The Different Protective Effects of COSe on Coelomocytes of Sea Cucumber Stimulated by LPS

#### 3.3.1. Protective Effect of COS on LPS-Stimulated Viability of Sea Cucumber Coelomocytes

**Figure 4:**
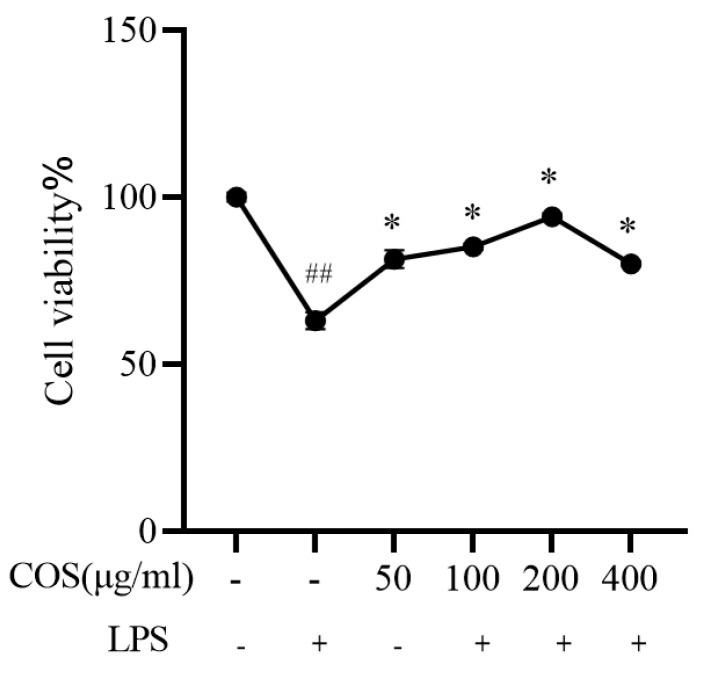
Protective effect of COS on LPS-stimulated coelomocytes. Cells were pre-treated with COS for 3 h and cultured in the presence of LPS for another 6 h. Cell viability in the absence or presence of LPS was determined by CCK-8 assay. * *p* < 0.05, ^##^ *p* < 0.01 when compared to the control group. + and - indicate the presence and absence of LPS or COS, respectively. Data are expressed as the means ± SD (*n* = 3).

Additionally, we used COS to treat coelomocytes to observe the protective effect on LPS-induced cell damage in vitro (Figure 4). The results showed that, compared with the control group, the activity of cells treated with LPS alone was significantly reduced by 36.76%. However, pre-treatment with COS led to a significant increase in cell viability compared to LPS alone, especially at COS concentrations of 100 and 200 μg/mL, a significant increase of 17.34% and 25.57%, respectively (*p* < 0.05). Based on these results, we selected a concentration of 100, 200 μg/mL for the subsequent cell experiments.

#### 3.3.2. Effect of COS on Phagocytic Index and Respiratory Burst Viability of LPS-Stimulated Sea Cucumber Coelomocytes

**Figure 5:**
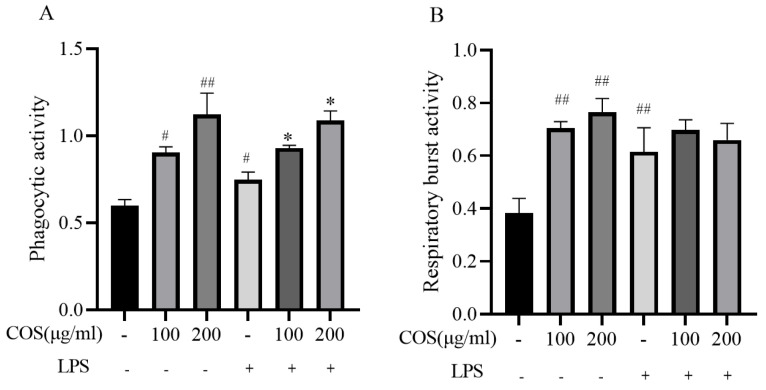
The effect of Chitosan oligosaccharides (COS) on phagocytic index (**A**) and respiratory burst viability (**B**) in LPS-stimulated sea cucumber coelomocytes. Data are expressed as the means ± SD (*n* = 3). * *p* < 0.05, ^#^ *p* < 0.05, ^##^ *p* < 0.01 when compared to the control group. + and - indicate the presence and absence of LPS or COS, and the numbers (100, 200) represent the addition of different concentrations of COS (μg/mL), respectively.

As shown in Figure 5A, chitosan oligosaccharide demonstrates a significant enhancement in the phagocytic activity of sea cucumber coelomocytes when tested in vitro. Specifically, concentrations of 100 and 200 μg/mL of chitosan oligosaccharide resulted in a notable increase in phagocytic activity, with the COS of 200 μg/mL group showing a remarkable 72.91% improvement compared to the control group (*p* < 0.05). Compared with the LPS alone treatment group, COS pre-incubation could significantly enhance the phagocytic activity of coelomocytes. Among them, 200 μg/mL COS has the most obvious reduction effect, which can significantly increase by 42.91% (*p* < 0.05).

Subsequently, the respiratory burst activity of the different treatment groups was analyzed (Figure 5B). Compared with the control group, LPS significantly increased the respiratory burst activity of coelomocytes by 20.55% (*p* < 0.05), while COS alone increased the respiratory burst activity of cells even more significantly than LPS. However, compared with LPS treatment, the respiratory burst activity of sea cucumber coelomocytes treated with LPS and COS increased, but not significantly.

#### 3.3.3. Effect of COS on Enzyme Activity of LPS-Stimulated Sea Cucumber Coelomocytes

**Figure 6:**
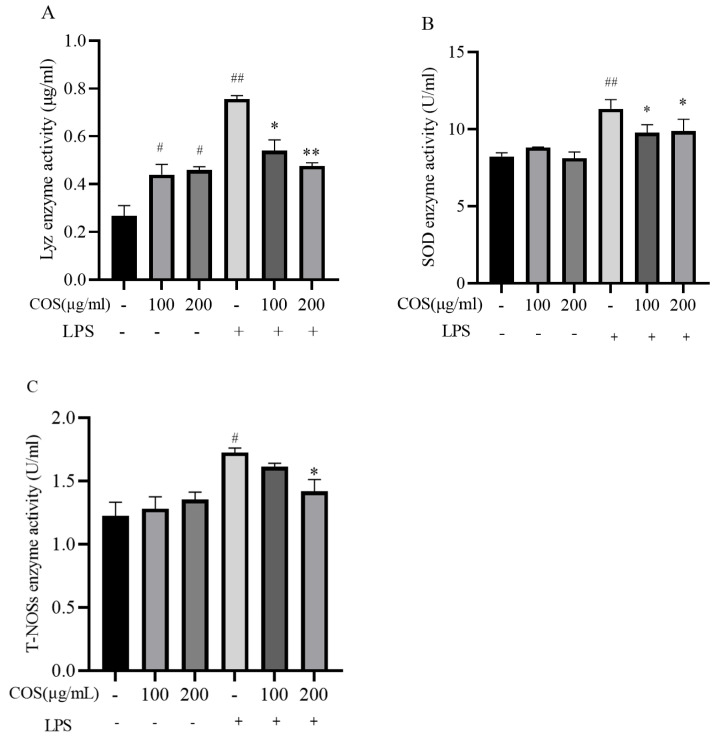
The effect of Chitosan oligosaccharides (COS) on enzyme activity in LPS-stimulated sea cucumber coelomocytes. Lyz (**A**), SOD (**B**), T-NOSs (**C**) were determined in LPS-stimulated sea cucumber coelomocytes that were treated with or without COS at concentrations of 100 or 200 μg/mL. Data are expressed as the means ± SD (*n* = 3). * *p* < 0.05, ** *p* < 0.01 when compared to the LPS alone group, ^#^ *p* < 0.05, ^##^ *p* < 0.01 when compared to the control group. + and - indicate the presence and absence of LPS or COS, and the numbers (100, 200) represent the addition of different concentrations of COS (μg/mL), respectively.

As shown in Figure 6, compared with the control group, whether 100 μg/mL COS or 200 μg/mL COS alone stimulated coelomocytes for 6 h, there was no significant change in the SOD enzyme and T-NOSs enzyme activities of coelomocytes (*p* > 0.05), but Lyz enzyme activity was significantly increased (*p* < 0.05). The LPS group can significantly increase the activities of various enzymes (*p* < 0.05). However, compared with the LPS group, the pre-incubation of coelomocyte with 200 μg/mL COS could significantly reduce the activity of SOD, Lyz, and NOS enzyme activities in coelomocytes (*p* < 0.05).

#### 3.3.4. Effect of COS on the Expression of Immune Genes of the Sea Cucumber Coelomocytes Stimulated by LPS

**Figure 7:**
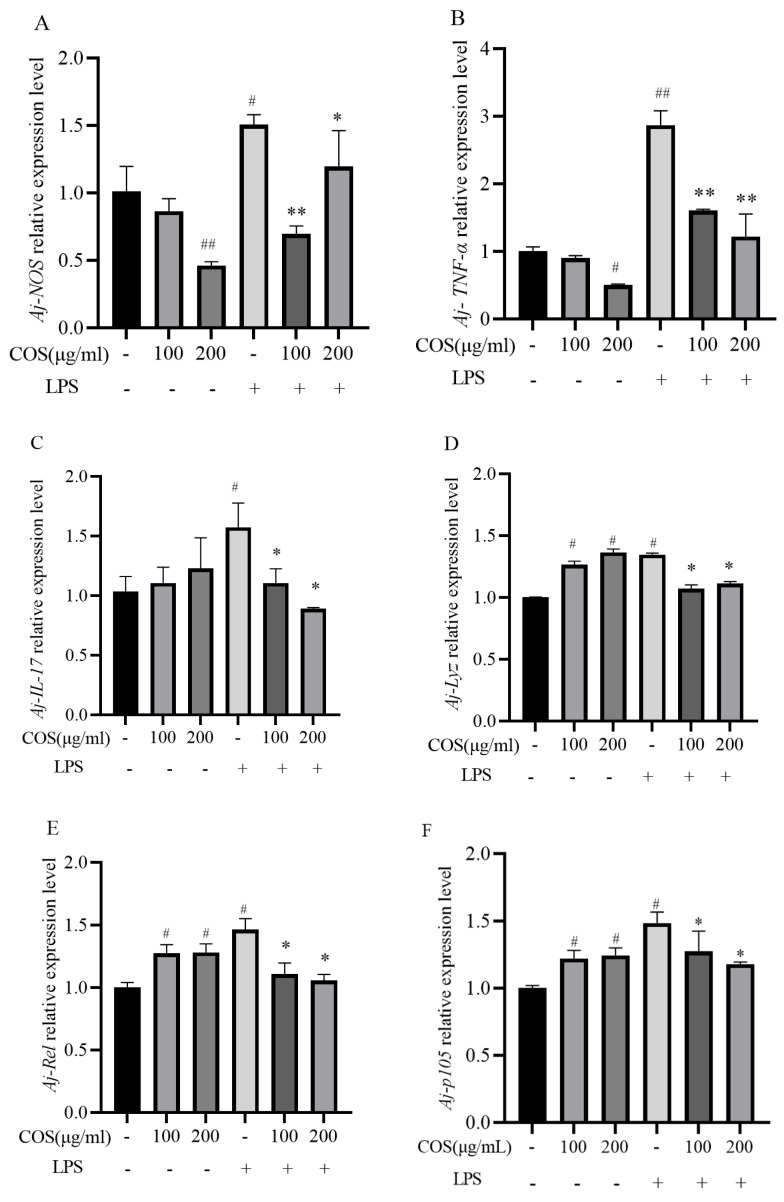
The effect of Chitosan oligosaccharides (COS) on gene expression in LPS-stimulated sea cucumber coelomocytes. The expression of genes related to pro-inflammatory markers was determined in LPS-stimulated sea cucumber coelomocytes that were treated with or without COS at concentrations of 100 or 200 μg/mL. The mRNA expression in the treated groups was normalized to that of in the negative control group (considered as 1) and is indicated as a relative value. (**A**) *Aj-NOS*, (**B**) *Aj-TNF-α*, (**C**) *Aj-IL-17*, (**D**) *Aj-Lyz*, (**E**) *Aj-Rel,* (**F**) *Aj-P105* relative expression levels. Data are expressed as the means ± SD (*n* = 3). * *p* < 0.05, ** *p* < 0.01 when compared to the LPS alone group, ^#^ *p* < 0.05, ^##^ *p* < 0.01 when compared to the control group. + and - indicate the presence and absence of LPS or COS, and the numbers (100, 200) represent the addition of different concentrations of COS (μg/mL), respectively.

As shown in Figure 7, after the cells were cultured with different proportions of COS and LPS, the mRNA relative expression levels of nine immune-related genes in the coelomic cells were detected by qPCR, respectively. Almost all the detected genes showed a similar trend of significant increase in expression levels after 6 h of LPS stimulation alone compared to the control group (*p* < 0.05). Among them, *Aj-IL-17*, *Aj-TNF-α*, *Aj-i-Lys*, *Aj-NOS*, *Aj-Rel*, and *Aj-P105* were up-regulated by 45.66%, 180.34%, 50.75%, 30.27%, 38.45%, and 37.47%, respectively. The pretreatment of cells with different concentrations of COS for 6 h in advance brought the expression of the gene back to normal levels. These results indicated that LPS could induce inflammatory response in coelomocytes, while COS could slow down the inflammatory response caused by LPS stimulation in cells.

### 3.4. The Result of Transcriptome Analysis

#### 3.4.1. Assessment of Transcriptome Quality

To obtain coelomocytes of *Apostichopus japonicus* stimulated by LPS, a total of six cDNA libraries were constructed, and each sample was repeated organically, including samples from the LPS and EG groups. Table 1 showed 427,158,145 clean data (98.50% < Q20 < 98.73%, 95.84% < Q30 < 96.68%) obtained by removing low-quality and unreliable sequences from 428,706,638 raw data, with the GC content of the 12 libraries ranging from 40.83% to 41.28%. The average fragment size in these libraries was between 149.37 and 149.56 bp (Table 2).

#### 3.4.2. Reference Sequence Alignment

The results are shown in Table 3. The average alignment rates of the two groups to the reference genome of *Apostichopus japonicus* were 69.64% and 69.35%, respectively, while the average uniquely mapped rate was no less than 61.61%, and the average multiple mapped rate was no higher than 7.73%. Notably, the percentage of sequencing reads aligned to exons in both groups exceeded 37.61%. Therefore, the sequencing results are reliable and conducive to further analysis in the next steps.

#### 3.4.3. Gene Expression Analysis

**Figure 8:**
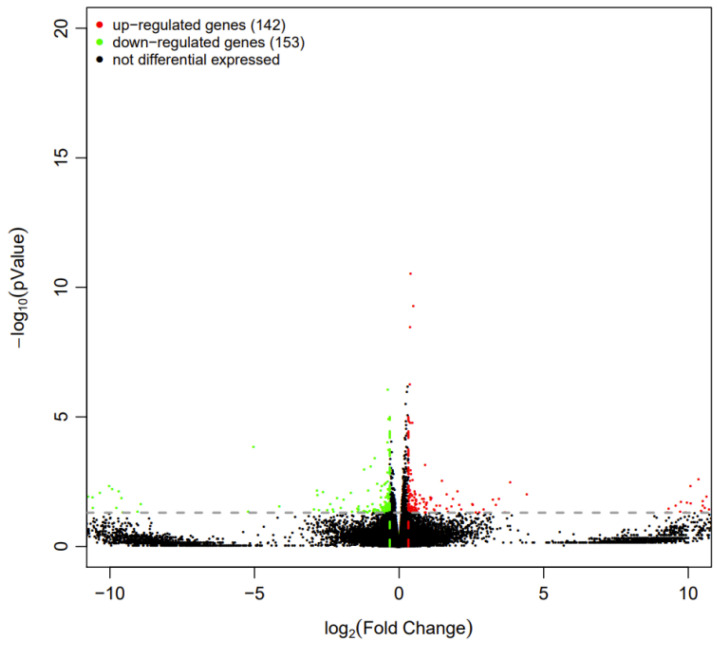
Volcano map of differentially expressed genes in coelomocytes of *Apostichopus japonicus.* Note: The x-axis represents the fold change in gene or transcript expression differences between the two groups, while the y-axis represents the statistical *p*-value of the gene or transcript expression differences. Each point in the plot represents a specific gene or transcript. Red points denote significantly upregulated genes, green points denote significantly downregulated genes, and black points denote genes with no significant differences.

Based on the negative binomial distribution, we utilized the DESeq2 tool to filter differentially expressed genes (DEGs) according to a *p*-value threshold of 1.25. Transcriptomic analyses indicated distinct differences in gene expression within the coelomocytes of *Apostichopus japonicus* when stimulated by LPS and chitosan oligosaccharide. A comparative analysis between the LPS group and the EG group revealed that, among the 295 DEGs identified, 142 genes were found to be upregulated, while 152 genes were downregulated (Figure 8).

#### 3.4.4. GO and KEGG Enrichment Analysis of DEGs

**Figure 9:**
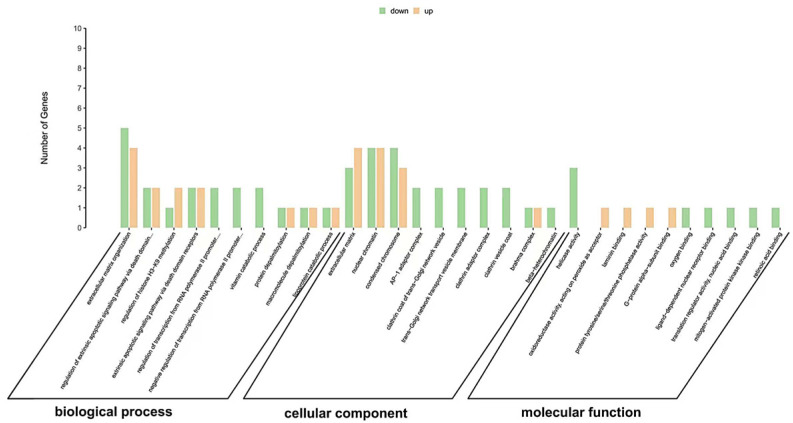
Gene Ontology (GO) annotations of different expression genes (DEGs).

To gain a deeper understanding of the functional distribution characteristics of differentially expressed genes in the coelomocytes of *Apostichopus japonicus*, we utilized the Gene Ontology (GO) database for the functional classification of these genes. Through GO enrichment analysis, we annotated a total of 75 significantly differentially expressed genes, which were categorized into three major categories: Biological Process (BP), Cellular Component (CC), and Molecular Function (MF). Specifically, the results of the GO enrichment analysis for upregulated and downregulated genes are illustrated in Figure 9. The downregulated genes were significantly enriched in extracellular matrix organization, while the upregulated genes were significantly enriched in extracellular matrix and nuclear chromatin.

**Figure 10:**
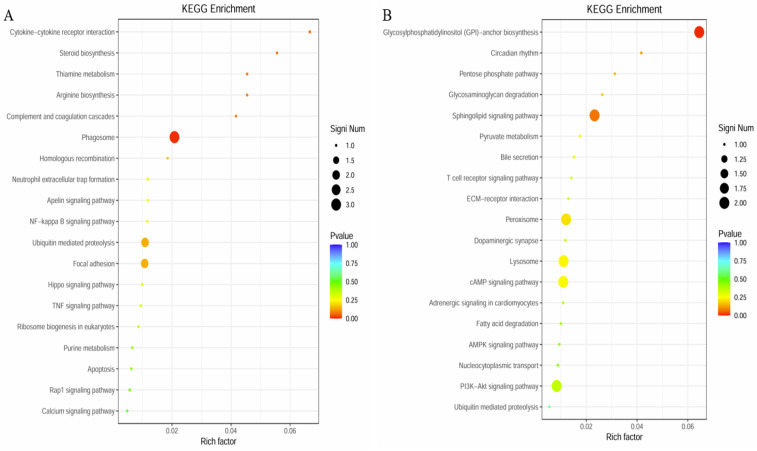
KEGG pathway enrichment of significantly differentially expressed genes. Note: (**A**): the upregulated pathways based on the DEGs of LPS vs EG group; (**B**): the downregulated pathways based on the DEGs of LPS vs EG group.

Bioinformatics analysis of gene functions based on the KEGG database assigned 295 DEGs to 55 pathways. We selected several significantly enriched pathways for display, as shown in Table 4. Among the downregulated genes in the KEGG enrichment pathways, two differentially expressed genes were associated with the Peroxisome, which was the highest in number and the most enriched; two genes were involved in the cAMP signaling pathway, showing significant enrichment; and two genes were related to the Lysosome, which was the most significantly enriched. In the upregulated gene KEGG enrichment pathways, there are three differentially expressed genes involved in phagosomes, which are the most numerous and have the highest enrichment level; there are two genes in the PI3K-Akt signaling pathway, showing significant enrichment with a high enrichment level; and there are two genes in focal adhesion, which are the most significantly enriched (Figure 10).

#### 3.4.5. qPCR Verification

**Figure 11:**
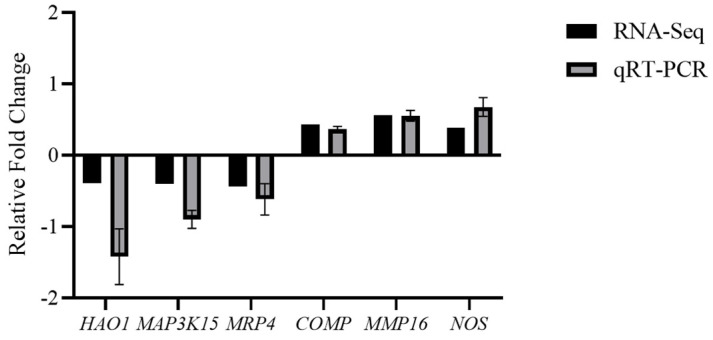
Validation of RNA-seq data by qRT-PCR.

In order to validate the transcriptomic results obtained from RNA-Seq, six specific genes (*HAO1*, *MRP4*, *MAP3K15*, *COMP*, *MMP16*, *NOS*) were selected for analysis using qPCR (Figure 11). The levels of mRNA expression, as assessed by q-PCR in conjunction with transcriptomic analysis, are illustrated in Figure 10. The findings from the q-PCR analysis corresponded closely with the RNA-Seq results, reinforcing the trustworthiness of the RNA-Seq data.

## 4. Discussion

In recent years, the role of COS as an emerging green non-toxic substance in immune enhancers has been gradually recognized. Our previous study showed that feeding COS in vivo could effectively improve the nonspecific immunity of the sea cucumber, could significantly increase the ability of the sea cucumber to resist *Vibrio splendidus* infection, and could improve the antioxidant capacity of sea cucumber coelomocytes [32]. LPS is a component of the outer membrane of gram-negative bacteria and is commonly used to induce an inflammatory response [33]. Pattern recognition receptors on the cell surface recognizes LPS, and binding to LPS leads to cell activation, resulting in the release of pro-inflammatory cytokines. Excessive pro-inflammatory cytokines lead to system disorders and multiple organ damage [34]. Currently, there are many applications of LPS to establish cellular inflammation models. For instance, the A549 cell line stimulated using LPS was applied as the inflammatory NSCLC model in vitro [35]. HK-2 cells stimulated with LPS were used to establish a cell model of AKI [36].

At present, there are a large number of literature reports focusing on the protective effect of COS on LPS-induced damage. For example, the inhibitive effects of COS on LPS-induced IL-6/TNF-α production in macrophages have been explored [37]. Moreover, Paiboon investigated the anti-inflammatory activity of COS using differentiated THP-1 cells, and they found that COS could reduce the production of multiple pro-inflammatory cytokines associated with LPS [38]. However, there is a lack of research on the immune protection of COS on sea cucumber coelomocytes in vitro.

In this study, the effects of COS on the LPS-induced immune responses of sea cucumber coelomocytes were observed. We investigated the respiratory burst, phagocytic activity, and other activities of four immune-related enzymes, namely ACP, AKP, CAT, and SOD, as well as the expression levels of six immune-related genes, namely *Aj-IL-17*, *Aj-TNF-α*, *Aj-i-Lys*, *Aj-NOS*, *Aj-Rel*, and *Aj-P50*, in the sea cucumber coelomocytes. The results showed that COS alone stimulated the coelomocytes of sea cucumber and could increase the respiratory burst and phagocytic activity of the cells, as well as the activities of immune-related enzymes and the expression levels of immune genes. COS could also reduce the cellular inflammatory response caused by the LPS stimulation of the cells and reduce the activities of immune enzymes and the expression levels of immune genes.

As an invertebrate, the sea cucumber lacks adaptive immunity and relies solely on innate immunity. Innate immunity encompasses cellular reactions such as phagocytosis and encapsulation, as well as humoral immunity, which produces immune-related factors [39]. Coelomocytes serve as the primary site for pathogen elimination in sea cucumbers [31,40]. Coelomic fluid is rich in hydrolytic enzymes, including lysozyme (LSZ), total nitric oxide synthase (T-NOSs), and superoxide dismutase (SOD), which could hydrolyze foreign pathogens [41,42]. Generally, the activity of immune-related enzymes is indicative of the host’s immune system status [32]. Lyz is an alkaline protein that can hydrolyze the cell wall released by bacterial lysis and form a hydrolytic enzyme system that can remove foreign bodies from the body and enhance the antibacterial properties of the body [43]. Studies have shown that feeding COS in vivo could significantly increase the lysozyme activity of sea cucumber coelomocytes [32]. In this study, the activity of lysozyme showed an increasing trend in coelomocytes treated with chitosan oligosaccharides (COS), with optimal activity observed at a concentration of 200 μg/mL. Conversely, Lyz activity exhibited a declining pattern in coelomocytes that were pretreated with COS and subsequently stimulated with lipopolysaccharides (LPS), which is consistent with the observed increase in *Aj-i-lyz* gene expression levels. It is hypothesized that lipopolysaccharide (LPS) effectively mimics the bacterial cell wall and binds to the receptors on the surface of sea cucumber body cavity cells, resulting in an increase in enzyme activity. However, the molecular mechanisms through which chitosan oligosaccharides (COS) attenuate LPS-mediated lysozyme activity warrant further investigation.

When the balance between reactive oxygen species (ROS) production and clearance is disrupted, oxidative stress occurs, leading to cellular damage [44]. Superoxide dismutase catalyzes the conversion of superoxide anion radicals (O^2−^) into oxygen (O^2^) and hydrogen peroxide (H_2_O_2_); as a crucial antioxidant enzyme, it serves as the first line of defense for organisms against oxygen toxicity [45]. Nitric oxide synthase (NOS) is a multidomain enzyme that catalyzes the production of nitric oxide (NO) by oxidizing l-Arg to NO and L-citrulline [46]. In this study, COS was demonstrated to reduce the activity of T-NOSs and SOD enzymes in LPS-stimulated sea cucumber coelomocytes in a dose-dependent manner, consistent with changes in the expression of the *Aj-NOS* gene. This finding indicated that COS simultaneously inhibited the activity of cellular immune enzymes and the expression of immune enzyme-related genes, thereby providing cellular protection.

The phagocytosis of coelomocytes is a crucial mechanism by which sea cucumbers engulf and eliminate foreign substances, serving as the primary line of defense within the organism [47]. Upon activation into a phagocytic state, coelomocytes undergo a respiratory burst, resulting in the production and release of significant quantities of superoxide anions and strong oxidants (O^2−^, OH^−^, O^2^, H_2_O_2_); these compounds play a vital role in extensive sterilization and the removal of foreign matter [48]. Studies have shown that the incubation of various chitosan oligosaccharide monomers enhanced phagocytosis activity toward *Staphylococcus aureus* and *Escherichia coli* and significantly boosted the generation of reactive oxygen species in RAW264.7 cells [49]. In this study, it was observed that, when COS alone stimulated sea cucumber coelomocytes, there was an enhancement in both the respiratory burst and phagocytic activity of the cells. Furthermore, COS appeared to enhance the respiratory burst and phagocytic activity of coelomocytes following LPS stimulation. It is speculated that COS might augment LPS-induced biomarkers in sea cucumber coelomocytes, leading to an increased production of reactive oxygen species (ROS) and a corresponding enhancement in the phagocytic activity of these coelomocytes.

In general, some immune-related regulatory or effector genes were significantly expressed to respond to the invasion of external changes and stimuli [5]. *TNF-α*, an important regulator of inflammation and immunity, could activate the NF-κB signaling pathway [50]. Interleukin-17 (*IL-17*) had an important role in autoimmune disorders and against bacterial, viral, and fungal pathogens [51]. Some research found that COS could inhibit the production of pro-inflammatory factors. For example, COS suppressed the expression of pro-inflammatory cytokines, such as *TNF-α* and *IL-6* in LPS-stimulated RAW264.7 macrophages, in a dose-dependent manner [52]. Moreover, the COS with a concentration of 100 mg/kg could reduce the elevation of inflammatory cytokines and serum biochemical indices caused by LPS, relieve the behavioral changes caused by LPS in mice, and improve the degree of damage to the liver and intestines of mice [34]. In this study, LPS significantly increased the secretion of inflammatory cytokines and the expression of *Aj-IL-17* and *Aj-TNF-α* at their mRNA levels. The cells treated with COS can effectively reduce the adverse effects of LPS in a concentration-dependent manner. These results suggest that chitosan may have the anti-inflammatory effect in LPS-stimulated inflammation. The NF-κB signaling pathway is considered a typical pro-inflammatory signaling pathway that regulates stress response, apoptosis, immunity, and differentiation. NF-κB plays an important role in the survival, activation, and differentiation of innate immune cells and in inducing the expression of pro-inflammatory mediators, including cytokines [53]. We subsequently examined whether COS affects the LPS-mediated activation of NF-κB in sea cucumber coelomocytes. Our results indicated that COS mitigates the adverse effects of LPS-stimulated cells by down-regulating the expression of *Aj-P105* and *Aj-Rel* genes. It has been reported that COS exerts an anti-inflammatory effect in macrophages by inhibiting the phosphorylation of MAPKs, NF-κB, and AP-1 [37]. Consequently, we will conduct further investigations to determine whether COS influences the immune activity of sea cucumber coelomocytes via the NF-κB signaling pathway.

Transcriptome technology in the study of aquatic animals helps to provide a theoretical basis for exploring the molecular mechanisms and molecular markers of growth, development, immunity, reproduction, metabolism, and genetics in aquatic organisms [54]. The experiment conducted transcriptome sequencing on coelomocytes stimulated by lipopolysaccharide after pre-incubation with chitosan, resulting in the annotation of 294 differentially expressed genes to 55 pathways. By combining the enrichment of upregulated and downregulated genes in KEGG, the most significantly enriched pathways were selected for the following analysis. In this experiment, the differentially expressed genes enriched in the phagosome signaling pathway (Phagosome) showed a significant upregulation of NOS. It is speculated that the Phagosome pathway may play an important role in the immunity of sea cucumbers, and these genes may be involved in the immune regulation of sea cucumber coelomocytes. NOS can catalyze the conversion of L-arginine to citrulline and NO. NO can stimulate macrophages to combat various target cells such as tumor cells, pathogenic bacteria, and mycobacteria. Therefore, NOS plays a significant role in both specific and non-specific immune defenses [55]. In this experiment, the NOS expression level was lower in the EG group, indicating that pre-incubation with COS could reduce the excessive production of NO induced by LPS and decrease ROS-mediated oxidative stress and inflammatory injury. The tumor necrosis factor (TNF) signaling pathway could induce a wide range of intracellular signaling pathways, including those related to cell apoptosis and cell survival as well as inflammation and immunity [56]. MMP16 has garnered attention for its involvement in various biological processes, including cancer metastasis, angiogenesis, and immune regulation [57]. In sea cucumbers, the research indicates that the *Aj-MMP-16* gene may play a significant role in the visceral regeneration, inflammation, and immune response of *Apostichopus japonicus*: after stimulation with *Vibrio splendidus*, the mRNA expression level of the MMP-16 gene in coelomocytes significantly increased [58]. In this study, the expression level of MMP16 in the coelomocyte EG group was significantly reduced, indicating that chitosan oligosaccharides can enhance the immune capacity of *Apostichopus japonicus* by acting on the tumor necrosis factor signaling pathway.

## 5. Conclusions

In summary, this study investigated the immunoprotective effects of COS on sea cucumber coelomocytes stimulated by LPS in vitro for the first time. The results demonstrated that a specific concentration of COS could significantly enhance both the respiratory burst and phagocytosis of sea cucumber coelomocytes. Moreover, the study revealed that COS positively influenced various immune indices, including the activity of immune-related enzymes and the expression levels of certain immune genes, thereby enhancing the immunity of coelomocytes in vitro. Additionally, COS appears to modulate the immune upregulation of sea cucumber coelomocytes in response to LPS stimulation. COS primarily alleviates LPS-induced inflammation via the tumor necrosis factor signaling pathway and the phagosome signaling pathway. This research offered novel insights into the role of COS in the immune response of sea cucumber coelomocytes following LPS exposure and served as a valuable reference for a deeper understanding of the innate immune response in sea cucumbers.

## Figures and Tables

**Table 1:** Primers used in this study.

Primer Name	Primer Sequence (5′-3′)
Aj-β-actin F	TGGCGTGAGGAAGAGCAT
Aj-β-actin R	CATTCAACCCTAAAGCCAACA
Aj-NOS F	GTAGAAGGAAAGGAGAGCGAGTC
Aj-NOS R	CATCGTGTCTCGTCGCATAGTGT
Aj-IL-17 F	GTTTGTGGTGCTGTTCTCTGTGA
Aj-IL-17 F	GGACTTCGATCGGGTCTTTTG
Aj-i-Lys F	CCTTACCAAATCAAACTAGGCTACTGG
Aj-i-Lys R	TAGGTTGCGTACCGTGCCATATAAC
Aj-TNF-α F	ACCCGACTCAACAACCAGAC
Aj-TNF-α R	ACACTGGACATTAGCAGGGC
Aj-Rel F	TGAAGGTGGTATGCGTCTGG
Aj-Rel R	TTGGGCTGCTCGGTTATG
Aj-P105 F	TCCTATCGGTCTGAATCTTCCAA
Aj-P105 R	TTTCTTCCCTTTCTGGCTATGTTC
HAO1 F	TAGAGGAAGTCGCAGAGGCT
HAO1 R	GGGTATCGACCGTCAGGAAC
MRP4 F	TCGGTCTGTTGCTCTCTTCG
MRP4 R	GATCCCTTGTAGGGCCAACC
COMP F	GCGGTCTACAACCCTCAACA
COMP R	GGGTTGTAATGGAAGGGGCA
MMP16 F	TGGCATGGGTTGGATAGACG
MMP16 R	CCGGAGACGATTCCAGTTCC
NOS F	TCAGACCTCATACCAGGGGG
NOS R	GCACAAGAACGCAAGGTGAG
MAP3K15 F	GGAGCCAGTATCCTTCGCTG
MAP3K15 R	CCGAGCTGTTATTCTCCGCC

**Table 2:** Statistics of transcriptomic sequences from the coelomocytes of *Apostichopus japonicus*.

Sample Name	Average Read Length (bp)	Raw Reads (n)	Raw Bases (G)	Clean Reads (n)	Clean Bases (G)	Q20 (%)	Q30 (%)	GC (%)
LPS1	149.48	57,217,954	6.84	57,009,954	6.21	98.55	96.09	41.23
LPS2	149.55	83,362,358	7.73	83,041,080	6.09	98.62	96.26	41.02
LPS3	149.56	53,115,034	6.08	52,902,128	6.32	98.67	96.17	40.83
EG1	149.37	76,442,714	7.67	76,203,248	7.41	98.73	96.68	41.28
EG2	149.44	79,620,204	7.82	79,306,896	7.38	98.50	95.84	41.26
EG3	149.43	78,948,374	7.72	78,694,839	7.29	98.49	96.28	41.27

**Table 3:** Sequencing alignment statistics.

Sample	Total Reads	Total Mapped	Uniquely Mapped	Mutiple Mapped	Non-Splice Read
LPS1	57,009,954	39,609,282 (69.75%)	35,394,962 (62.33%)	4,214,320 (7.42%)	22,029,746 (38.80%)
LPS2	83,041,080	57,725,743 (69.72%)	51,467,320 (62.17%)	6,258,423 (7.56%)	32,287,292 (39.00%)
LPS3	52,902,128	36,627,260 (69.46%)	32,775,596 (62.15%)	3,851,664 (7.30%)	20,872,019 (39.58%)
EG1	76,203,248	52,224,656 (68.75%)	46,160,824 (60.76%)	6,063,832 (7.98%)	28,569,948 (37.61%)
EG2	79,306,896	55,560,044 (70.25%)	49,481,416 (62.56%)	6,078,628 (7.69%)	30,666,025 (38.77%)
EG3	98,759,504	67,936,435 (69.05%)	60,532,903 (61.52%)	7,403,532 (7.52%)	37,309,273 (37.92%)

**Table 4:** The differentially expressed genes involved in key KEGG pathways.

Gene ID	Gene Name	Gene Description	Fold Change
	Peroxisome		
BSL78_06822	*HAO1*	putative hydroxyacid oxidase 1	−0.395
BSL78_14742	*HAO1*	putative hydroxyacid oxidase 1	−0.394
	Lysosome		
BSL78_08883	*BSL78_08883*	hypothetical protein	−2.769
BSL78_10452	*BSL78_10452*	hypothetical protein	−0.322
	PI3K-Akt signaling pathway		
BSL78_19875	*BSL78_19875*	putative tenascin isoform X1	−10.977
BSL78_21813	*PP2A*	putative serine/threonine-protein phosphatase 2A regulatory subunit B″ subunit gamma	−12.589
BSL78_01699	*COMP*	putative cartilage oligomeric matrix protein	0.429
BSL78_06396	*BSL78_06396*	hypothetical protein	0.326
	cAMP signaling pathway		
BSL78_27675	*MRP4*	putative multidrug resistance-associated protein 4-like	−0.441
BSL78_21913	*BSL78_21913*	hypothetical protein	−0.839
	TNF signaling pathway		
BSL78_15356	*MMP16*	Matrix metalloproteinase-16	0.557
BSL78_04534	*MAP3K15*	putative mitogen-activated protein kinase kinase kinase 15-like	−0.406
	Phagosome		
BSL78_26181	*NOS*	putative nitric oxide synthase, brain isoform X2	0.387
BSL78_01699	*COMP*	putative cartilage oligomeric matrix protein	0.429

## Data Availability

The datasets used and/or analyzed during the current study are available from the corresponding author upon reasonable request (due to privacy).
